# Supplementary material for: Drosophila larval to pupal switch under nutrient stress requires IP3R/Ca2+ signalling in glutamatergic interneurons
Source: eLife. 2016 Aug 5;5:e17495. doi: 10.7554/eLife.17495 (PMC4993588; doi:10.7554/eLife.17495)
Supplement: Supplementary file 1. — DOI: http://dx.doi.org/10.7554/eLife.17495.028 [file elife-17495-supp1.docx]

List of Fly stocks

| **Fly line** | **Description** | **Source** |
| --- | --- | --- |
| *itpr^ug3^* | *itpr* Mutant | (Joshi et al., 2004) |
| *itpr^ka1091^* | *itpr* Mutant | (Joshi et al., 2004) |
| *Dilp2mCherry* | mCherry is expressed under Dilp2 promoter | Gift from Amita Sehgal (Cavanaugh et al., 2014) |
| *elav-GAL4* | Pan-neuronal driver | (Luo et al., 1994) |
| *OK371-GAL4* | Glutamatergic driver | (Mahr and Aberle, 2006) |
| *VGN6341-GAL4* | Subset Glutamatergic driver | (Syed et al., 2015) |
| *VGN9281(2)-GAL4* | Subset Glutamatergic driver | (Sadaf et al., 2015) |
| *C929(dimm)-GAL4* | Peptidergic driver | (Hewes et al., 2003) |
| *ChAT-GAL4* | Cholinergic driver | (Salvaterra and Kitamoto, 2001) |
| *Tdc2-GAL4* | Octopaminergic driver | BL9313 |
| *BL-GAL4* | Brain Lobe driver | Gift from Michael Bate  (Berni et al., 2012) |
| *ppk-GAL4* | Class IV multidendritic driver (very weak expression in class III) | BL32078  (Zhong et al., 2010) |
| *19-12-GAL4* | Class III multidendritic driver | Gift from Yuh Nung Jan  (Yan et al., 2013) |
| *Dilp2-GAL4* | Dilp2 driver | (Rulifson et al., 2002) |
| *tsh-GAL80* | Inhibits GAL4 in ventral ganglion (under teashirt promoter) | Gift from Julie Simpson  (Albin et al., 2015; Clyne and Miesenböck, 2008) |
| *ppk-GAL80* | inhibits GAL4 in class IV multidendtritic cells | Gift from Yuh Nung Jan  (Yang et al., 2009) |
| *UAS-itpr IR* | RNAi line for *itpr* | 1063-R2 from NIG |
| *UAS-mAcR IR* | RNAi line for mAcR | VDRC 101407 CG4356  (Agrawal et al., 2013) |
| *UAS-itpr^+^* | Expresses *itpr* | (Venkatesh et al., 2001) |
| *UAS-mAcR^+^* | Expresses *mAcR* | (Venkiteswaran and Hasan, 2009) |
| *UAS-Kir2.1* | Inhibitor of neuronal activity | (Baines et al., 2001) |
| *UAS-mcd8GFP* | GFP in membranes | BL5130 |
| *UAS-H_2_BmRFP* | GFP in nuclei | Gift from Boris Egger  (Langevin et al., 2005) |
| *UAS-eGFP* | Cytosolic GFP | Gift from Michael Rosbash |
| *UAS-Shi^ts^* | Inhibits vesicle recycling | (Salvaterra and Kitamoto, 2001) |
| *UAS-mGluR IR* | RNAi for mGluR_A_ | CG11144 Trip Line BL 41668 |
| *UAS-mGluR IR2* | RNAi for mGluR_A_ | CG11144 v103736, VDRC |
| *UAS-NR1 IR* | RNAi for NR1 | CG2902 v373334, VDRC |
| *UAS-GluR2 IR* | RNAi for GluR2 | CG6992 v101686VDRC |
| *UAS-GluR1B IR* | RNAi for GluR1B | CG4481 v42890, VDRC |
| *UAS-GluR1 IR* | RNAi for GluR1 | CG8442 v44438, VDRC |
| *UAS-GCaMP6m* | Genetically encoded Calcium indicator | BL42748 |
| *UAS-jRCaMP1b* | Red shifted Calcium indicator | BL63793  (Dana et al., 2016) |
| *UAS-dTrpA_1_* | Cation channel TrpA1 | BL26263  (Hamada et al., 2008) |
| *UAS-NachBac* | Bacterial sodium Channel | BL9468  (Nitabach et al., 2006) |
| *UAS-CsChrimson* | Red shifted optogenetic activator | BL 55135  (Klapoetke et al., 2014) |
| *UAS-eNpHR2* | Optogenetic inhibitor | Gift from Leslie Griffith  (Berni et al., 2012) |
| *UAS-dilp2^+^* | Expresses *Dilp2* | (Brogiolo et al., 2001) |
| *UAS-syteGFP;UAS-Denmark* | Both axonal and dendritic marker | BL33065  (Nässel et al., 2008; Nicolai et al., 2010) |
| *UAS-ANF::GFP* | Expresses ANF::GFP | BL 7001  (Shakiryanova et al., 2006) |
| *UAS-AcGq* | Constitutive form of *Gq* | (Ratnaparkhi et al., 2002) |
| *UAS-dSTIM* | Expresses *dSTIM* | (Agrawal et al., 2010) |
| *LexAop-CD4::GFP11; UAS-CD4::GFP1-10* | GRASP | Gift from Kristin Scott  (Gordon and Scott, 2009) |
| *HL9-LexA* | Aminergic driver | Gift from Yoshi Aso |
| *ChAT-LexA* | Cholinergic driver | (Diegelmann et al., 2008) |
| *LexAop-GCaMP6m* | Genetically encoded calcium indicator | BL44276  (Chen et al., 2013) |
| *LexAop-CsChrimson* | Red Shifted optogenetic activator | BL55138  (Klapoetke et al., 2014) |
| *LexAop-mCherry* | Expresses mCherry under LexA Control | Gift from Claude Desplan |
| *ppk-QF* | Class IV multidendritic driver (very weak expression in class III) | BL36348  (Petersen and Stowers, 2011) |
| *QUAS-mCherry* | Expresses mCherry under QF control | BL52270  (Petersen and Stowers, 2011) |
| *QUAS-ChR2* | Expresses channelrhodopsin under QF control | BL58400  (Liang et al., 2013) |

**References**

Agrawal, N., Venkiteswaran, G., Sadaf, S., Padmanabhan, N., Banerjee, S., and Hasan, G. (2010). Inositol 1,4,5-Trisphosphate Receptor and dSTIM Function in Drosophila Insulin-Producing Neurons Regulates Systemic Intracellular Calcium Homeostasis and Flight. J. Neurosci. *30*, 1301–1313.

Agrawal, T., Sadaf, S., and Hasan, G. (2013). A genetic RNAi screen for IP₃/Ca^2+^ coupled GPCRs in Drosophila identifies the PdfR as a regulator of insect flight. PLoS Genet. *9*, e1003849.

Albin, S.D., Kaun, K.R., Knapp, J.-M., Chung, P., Heberlein, U., and Simpson, J.H. (2015). A Subset of Serotonergic Neurons Evokes Hunger in Adult Drosophila. Curr. Biol. *25*, 2435–2440.

Baines, R. a, Uhler, J.P., Thompson, a, Sweeney, S.T., and Bate, M. (2001). Altered electrical properties in Drosophila neurons developing without synaptic transmission. J. Neurosci. *21*, 1523–1531.

Berni, J., Pulver, S.R., Griffith, L.C., and Bate, M. (2012). Autonomous circuitry for substrate exploration in freely moving drosophila larvae. Curr. Biol. *22*, 1861–1870.

Brogiolo, W., Stocker, H., Ikeya, T., Rintelen, F., Fernandez, R., and Hafen, E. (2001). An evolutionarily conserved function of the drosophila insulin receptor and insulin-like peptides in growth control. Curr. Biol. *11*, 213–221.

Cavanaugh, D.J., Geratowski, J.D., Wooltorton, J.R.A., Spaethling, J.M., Hector, C.E., Zheng, X., Johnson, E.C., Eberwine, J.H., and Sehgal, A. (2014). Identification of a circadian output circuit for rest: Activity rhythms in drosophila. Cell *157*, 689–701.

Chen, T.-W., Wardill, T.J., Sun, Y., Pulver, S.R., Renninger, S.L., Baohan, A., Schreiter, E.R., Kerr, R. a, Orger, M.B., Jayaraman, V., et al. (2013). Ultrasensitive fluorescent proteins for imaging neuronal activity. Nature *499*, 295–300.

Clyne, J.D., and Miesenböck, G. (2008). Sex-Specific Control and Tuning of the Pattern Generator for Courtship Song in Drosophila. Cell *133*, 354–363.

Dana, H., Mohar, B., Sun, Y., Narayan, S., Gordus, A., Hasseman, J.P., Tsegaye, G., Holt, G.T., Hu, A., Walpita, D., et al. (2016). Sensitive red protein calcium indicators for imaging neural activity. Elife *5*, e12727.

Diegelmann, S., Bate, M., and Landgraf, M. (2008). Gateway cloning vectors for the LexA-based binary expression system in drosophila. Fly (Austin). *2*, 236–239.

Gordon, M.D., and Scott, K. (2009). Motor control in a Drosophila taste circuit. Neuron *61*, 373–384.

Hamada, F.N., Rosenzweig, M., Kang, K., Pulver, S.R., Ghezzi, A., Jegla, T.J., and Garrity, P. a (2008). An internal thermal sensor controlling temperature preference in Drosophila. Nature *454*, 217–220.

Hewes, R.S., Park, D., Gauthier, S. a, Schaefer, A.M., and Taghert, P.H. (2003). The bHLH protein Dimmed controls neuroendocrine cell differentiation in Drosophila. Development *130*, 1771–1781.

Joshi, R., Venkatesh, K., Srinivas, R., Nair, S., and Hasan, G. (2004). Genetic Dissection of itpr Gene Function Reveals a Vital Requirement in Aminergic Cells of Drosophila Larvae. Genetics *166*, 225–236.

Klapoetke, N.C., Murata, Y., Kim, S.S., Pulver, S.R., Birdsey-Benson, A., Cho, Y.K., Morimoto, T.K., Chuong, A.S., Carpenter, E.J., Tian, Z., et al. (2014). Independent optical excitation of distinct neural populations. Nat. Methods *11*, 338–346.

Langevin, J., Le Borgne, R., Rosenfeld, F., Gho, M., Schweisguth, F., and Bella??che, Y. (2005). Lethal giant larvae controls the localization of Notch-signaling regulators Numb, neuralized, and Sanpodo in Drosophila sensory-organ precursor cells. Curr. Biol. *15*, 955–962.

Liang, L., Li, Y., Potter, C., Yizhar, O., Deisseroth, K., Tsien, R., and Luo, L. (2013). GABAergic Projection Neurons Route Selective Olfactory Inputs to Specific Higher-Order Neurons. Neuron *79*, 917–931.

Luo, L., Joyce Liao, Y., Jan, L.Y., and Jan, Y.N. (1994). Distinct morphogenetic functions of similar small GTPases: Drosophila Drac1 is involved in axonal outgrowth and myoblast fusion. Genes Dev. *8*, 1787–1802.

Mahr, A., and Aberle, H. (2006). The expression pattern of the Drosophila vesicular glutamate transporter: A marker protein for motoneurons and glutamatergic centers in the brain. Gene Expr. Patterns *6*, 299–309.

Nässel, D.R., Enell, L.E., Santos, J.G., Wegener, C., and Johard, H.A.D. (2008). A large population of diverse neurons in the Drosophila central nervous system expresses short neuropeptide F, suggesting multiple distributed peptide functions. BMC Neurosci. *9*, 90.

Nicolai, L.J.J., Ramaekers, A., Raemaekers, T., Drozdzecki, A., Mauss, A.S., Yan, J., Landgraf, M., Annaert, W., and Hassan, B.A. (2010). Genetically encoded dendritic marker sheds light on neuronal connectivity in Drosophila. Proc. Natl. Acad. Sci. *107*, 20553–20558.

Nitabach, M.N., Wu, Y., Sheeba, V., Lemon, W.C., Strumbos, J., Zelensky, P.K., White, B.H., and Holmes, T.C. (2006). Electrical hyperexcitation of lateral ventral pacemaker neurons desynchronizes downstream circadian oscillators in the fly circadian circuit and induces multiple behavioral periods. J. Neurosci. *26*, 479–489.

Petersen, L.K., and Stowers, R.S. (2011). A Gateway MultiSite Recombination Cloning Toolkit. PLoS One *6*, e24531.

Ratnaparkhi, A., Banerjee, S., and Hasan, G. (2002). Altered levels of Gq activity modulate axonal pathfinding in Drosophila. J. Neurosci. *22*, 4499–4508.

Rulifson, E.J., Kim, S.K., and Nusse, R. (2002). Ablation of insulin-producing neurons in flies: growth and diabetic phenotypes. Science *296*, 1118–1120.

Sadaf, S., Reddy, O.V., Sane, S.P., and Hasan, G. (2015). Report Neural Control of Wing Coordination in Flies. Curr. Biol. *25*, 80–86.

Salvaterra, P.M., and Kitamoto, T. (2001). Drosophila cholinergic neurons and processes visualized with Gal4/UAS-GFP. Gene Expr. Patterns *1*, 73–82.

Shakiryanova, D., Tully, A., and Levitan, E.S. (2006). Activity-dependent synaptic capture of transiting peptidergic vesicles. Nat. Neurosci. *9*, 896–900.

Syed, D.S., Gowda, S.B.M., and Reddy, O.V. (2015). Glial and neuronal Semaphorin signaling instruct the development of a functional myotopic map for. 1–18.

Venkatesh, K., Siddhartha, G., Joshi, R., Patel, S., and Hasan, G. (2001). Interactions between the inositol 1,4,5-trisphosphate and cyclic AMP signaling pathways regulate larval molting in Drosophila. Genetics *158*, 309–318.

Venkiteswaran, G., and Hasan, G. (2009). Intracellular Ca2? signaling and store-operated Ca2? entry are required in neurons for flight. *2009*.

Yan, Z., Zhang, W., He, Y., Gorczyca, D., Xiang, Y., Cheng, L.E., Meltzer, S., Jan, L.Y., and Jan, Y.N. (2013). Drosophila NOMPC is a mechanotransduction channel subunit for gentle-touch sensation. Nature *493*, 221–225.

Yang, C.H., Rumpf, S., Xiang, Y., Gordon, M.D., Song, W., Jan, L.Y., and Jan, Y.N. (2009). Control of the Postmating Behavioral Switch in Drosophila Females by Internal Sensory Neurons. Neuron *61*, 519–526.

Zhong, L., Hwang, R.Y., and Tracey, W.D. (2010). Pickpocket Is a DEG/ENaC Protein Required for Mechanical Nociception in Drosophila Larvae. Curr. Biol. *20*, 429–434.
