# Supplementary material for: Drosophila larval to pupal switch under nutrient stress requires IP3R/Ca2+ signalling in glutamatergic interneurons
Source: eLife. 2016 Aug 5;5:e17495. doi: 10.7554/eLife.17495 (PMC4993588; doi:10.7554/eLife.17495)
Supplement: Supplementary file 2. — DOI: http://dx.doi.org/10.7554/eLife.17495.029 [file elife-17495-supp2.docx]

Primer Sequences:

| Name | Sequence 5’>3’ (Annealing regions are capitalized) |
| --- | --- |
| *dimmed_F* | tatttcatagtcgacgatttttgttcagccatcggaaggcttcaacaatcATGCCACCCAAGAAGAAGCG |
| *dimmed_R* | aatggaaggcattataataggcccatttgtttggccttcttacgatctacGATCTAAACGAGTTTTTAAGCAAACTCACTCCC |
| *VGN6341-LexA_F* | ggggacaagtttgtacaaaaaagcaggcttaTTTTCGCCTTTTTGCAGTC |
| *VGN6341-LexA_R* | ggggacaacttttgtatacaaagttgtGCTTCAGCAGCAAACAATGA |
| *LexAop-ANF_F* | ggggacaactttgtatacaaaagttgtaATGGGCTCCTTCTCCATCAC |
| *LexAop-ANF_R* | ggggaccactttgtacaagaaagctgggttTTACTTGTACAGCTCGTCCATG |
| *rp49_F* | CGGATCGATATGCTAAGCTGT |
| *rp49_R* | GCGCTTGTTCGATCCGTA |
| *Dilp2_F* | CCATGAGCAAGCCTTTGTCC |
| *Dilp2_R* | TTCACTGCAGAGCGTTCCTTG |
